# Targeting IL-6 by both passive or active immunization strategies prevents bleomycin-induced skin fibrosis

**DOI:** 10.1186/ar4672

**Published:** 2014-07-24

**Authors:** Lucille Desallais, Jérôme Avouac, Maxime Fréchet, Muriel Elhai, Rojo Ratsimandresy, Matthieu Montes, Hadley Mouhsine, Hervé Do, Jean-François Zagury, Yannick Allanore

**Affiliations:** Chaire de Bioinformatique, Laboratoire Génomique, Bioinformatique et Applications, EA 4627, Conservatoire National des Arts et Métiers, 292 Rue Saint-Martin, 75003 Paris, France; Université Paris Descartes, Sorbonne Paris Cité, Service de Rhumatologie A, Hôpital Cochin, Université Paris Descartes, 27 rue du Faubourg Saint-Jacques, 75014 Paris, France; INSERM U1016/CNRS UMR8104, Institut Cochin, 22 Rue Mechain, 75014 Paris, France; Peptinov, Cochin Santé Business Accelerator (“Pépinière Cochin Santé”), Cochin Hospital, 29 rue du Faubourg Saint Jacques, 75014 Paris, France

## Abstract

**Introduction:**

Interleukin-6 (IL-6) is a pleiotropic cytokine for which preliminary data have suggested that it might contribute to systemic sclerosis (SSc). Our aims were to investigate, firstly, IL-6 expression in patients with SSc and, secondly, the efficacy of both passive and active immunization against IL-6 to reduce skin fibrosis in complementary mouse models of SSc.

**Methods:**

Human serum levels and skin expression of IL-6 were determined by enzyme-linked immunosorbent assay and immunohistochemistry, respectively. We first evaluated the antifibrotic properties of the monoclonal anti-IL-6R antibody, MR16-1, in the bleomycin-induced dermal fibrosis mouse model, reflecting early and inflammatory stages of SSc. Then, we assessed the efficacy of MR16-1 in tight skin-1 (Tsk-1) mice, an inflammation-independent model of skin fibrosis. Additionally, we have developed an innovative strategy using an anti-IL-6 peptide-based active immunization. Infiltrating leukocytes, T cells, and B cells were quantified, and IL-6 levels were measured in the serum and lesional skin of mice after passive or active immunization.

**Results:**

Serum and skin levels of IL-6 were significantly increased in patients with early SSc. Treatment with MR16-1 led in the bleomycin mouse model to a 25% (*P* = 0.02) and 30% (*P* = 0.007) reduction of dermal thickness and hydroxyproline content, respectively. MR16-1 demonstrated no efficacy in Tsk-1 mice. Thereafter, mice were immunized against a small peptide derived from murine IL-6 and this strategy led in the bleomycin model to a 20% (*P* = 0.02) and 25% (*P* = 0.005) decrease of dermal thickness and hydroxyproline content, respectively. Passive and active immunization led to decreased T-cell infiltration in the lesional skin of mice challenged with bleomycin. Upon bleomycin injections, serum and skin IL-6 levels were increased after treatment with MR16-1 and were significantly reduced after anti-IL-6 active immunization.

**Conclusions:**

Our results support the relevance of targeting IL-6 in patients with early SSc since IL-6 is overexpressed in early stages of the disease. Targeting IL-6 by both passive and active immunization strategies prevented the development of bleomycin-induced dermal fibrosis in mice. Our results highlight the therapeutic potential of active immunization against IL-6, which is a seductive alternative to passive immunization.

## Introduction

Systemic sclerosis (SSc, scleroderma) is a connective tissue disease of unknown etiology that affects particularly the skin. Early stages of SSc are characterized by vascular changes and inflammatory infiltrates in the lesional skin [1]. Later stages of SSc are characterized by an excessive accumulation of extracellular matrix components, including collagen, leading to increased skin thickness.

Several lines of evidence suggest a pathologic role of cytokine overproduction in the pathogenesis of SSc, particularly in fibroblast activation, collagen synthesis, and subsequent fibrosis. Interleukin-6 (IL-6) is a pleiotropic cytokine whose activities stimulate the proliferation and differentiation of B and T lymphocytes, enhance antibody production, activate T cells, stimulate hematopoietic precursors to differentiate, influence the proliferative capacity of non-lymphoid cells, and activate acute-phase protein response [2]. Preliminary data suggest that IL-6 might contribute to human SSc: levels of IL-6 are increased in the serum and in the lesional skin of patients with SSc, spontaneous production of IL-6 by peripheral blood leukocytes from patients with SSc is elevated compared with healthy controls, and IL-6 levels correlate with skin thickness score [3–12]. In addition, two preliminary reports have showed that passive immunization with anti-IL-6 receptor (IL-6R) monoclonal antibody may alleviate skin disease in two mouse models of inflammation-driven dermal fibrosis [13, 14]. However, the anti-fibrotic properties of IL-6 inhibition have not yet been assessed in mouse models of SSc that reflect later and non-inflammatory stages of SSc. Moreover, molecular targeted inhibition of IL-6 signaling *in vivo* was restricted to passive immunization, which may present several drawbacks, including primary and secondary resistances, repeated injections, side effects, and prohibitive costs. As an alternative and innovative strategy, our group has developed peptide-based anti-cytokine active immunization, which consists in inducing autoantibodies through an immunization against peptides of cytokines linked to a carrier protein (for example, keyhole limpet hemocyanin, or KLH) [15–17]. This promising strategy has not been used so far for IL-6 but has been successfully established for other cytokines, including tumor necrosis factor-alpha (TNFα) and IL-1β and IL-23 in different autoimmune diseases [15–18]. Therefore, in this study, our aim was to compare the antifibrotic properties of both passive and active immunization against IL-6 in complementary mouse models of SSc.

## Materials and methods

### Human skin biopsies

Paraffin-embedded sections of lesional skin biopsies were obtained from 10 patients with SSc and five healthy age- and sex-matched healthy volunteers. The median age of patients with SSc (eight females and two males) was 55 years (range 39 to 65 years), and disease duration was 4.5 years (range 1 to 12 years). Five patients with SSc had a disease duration of less than 5 years; four had the diffuse cutaneous subset, and six had the limited. No patient was treated with immunosuppressive or other potentially disease-modifying drugs. The median age of controls (four females and one male) was 57 years (range 31 to 62 years). All of the study aspects were approved by the local ethics review committee (Comité Consultatif de Protection des Personnes dans la Recherche Biomédicale Paris Ile de France III), and written informed consent was obtained from all patients and controls [9, 19, 20].

### Animals

Four- and six-week-old male and female DBA/2 strain (Janvier, Le Genest Saint Isle, France) and five-week-old male and female tight skin-1 (Tsk-1) strain (The Jackson Laboratory, Bar Harbor, ME, USA) were bred and maintained at the animal care facilities of Montrouge dental university (Montrouge, France). All experimental procedures were conducted in compliance with animal health regulations, and the local ethics committee approved all animal experiments (Comité National de Réflexion Ethique sur l’Expérimentation Animale-34).

### Induction of bleomycin-induced dermal fibrosis

Skin fibrosis was induced in 6-week-old DBA/2 mice by administering local injections of bleomycin for 3 weeks (d0 to d21), as previously described [8, 9, 21]. Briefly, 100 μL of bleomycin dissolved in 0.9% NaCl at a concentration of 0.5 mg/mL was administered every other day by subcutaneous injection into defined areas of 1 cm^2^ on the upper back for 3 weeks. Subcutaneous injections of 100 μL of 0.9% NaCl were used as negative control.

### Tight skin mouse model

The Tsk-1 phenotype is caused by a dominant mutation in the fibrillin-1 gene [22]. Tsk-1 mice are characterized by accumulation of collagen fibers in the hypodermis resulting in progressive hypodermal thickening. In contrast to bleomycin-induced fibrosis, inflammatory infiltrates are absent and the aberrant activation of fibroblasts is not caused by the release of inflammatory mediators from leukocytes. Similar to SSc fibroblasts, fibroblasts from Tsk-1 are endogenously activated with increased release of collagen that persists for several passages *in vitro*.

### Anti-IL-6 receptor monoclonal antibody treatment

Rat anti-mouse IL-6 receptor monoclonal antibody (clone MR16-1) described previously was provided by Chugai Pharmaceutical (Tokyo, Japan) [13, 23]. Purified rat IgG1 (isotype-matched control antibody, MP Biomedicals, Illkirch, France) was administered as a negative control. DBA/2 mice received, in parallel of bleomycin injections, an intraperitoneal (i.p.) injection of 2 mg at day 0 followed by one i.p. injection of 1 mg at days 7 and 14. Mice were sacrificed at day 21. Tsk-1 mice received a first i.p. injection of 2 mg at the age of 5 weeks followed by one i.p. injection of 1 mg once a week. Mice were sacrificed by cervical dislocation at the age of 10 weeks.

### Selection of the peptide derivative of mouse IL-6

At the beginning of the study, no structural data on mouse IL-6 were available. Peptide was designed by using the human IL-6/IL-6Rα/gp130 structure (Protein Data Bank (PDB) code: 1P9M). We could assume that the chosen site was transposable to mIL-6 because of the high conservation between mIL-6 and hIL-6 amino acid sequences. This hypothesis was verified after the publication of the mIL-6 structure in 2012 (PDB code: 2L3Y). Peptide was chosen in a loop exposed to the protein surface, which corresponded to a region involved in the interaction between the cytokine IL-6 and its receptor IL-6Rα.

### Peptides synthesis and cyclisation

Peptides were synthesized by PolyPeptide Laboratories (Strasbourg, France). Peptides were analyzed by high-pressure liquid chromatography and mass spectrometry. Only fractions with purity superior to 85% were conserved. mIS200 peptide was produced in a cyclized form by the formation of intramolecular disulphide bonds between cysteine residues.

### Coupling of the peptide to the carrier protein

To ensure a specific coupling of the mIS200 peptide to KLH, an additional tyrosine residue was added at its C-terminus extremity before proceeding to the coupling with bis-diazobenzidine (BDB). Briefly, the free amines of the peptide were protected with citraconic acid at pH 8.5 to 9. Then, the peptide, the KLH, and a solution of BDB were mixed together in a borate buffer for 2 hours at 4°C. The coupled peptide was then submitted to two dialyses against 5% acetic acid followed by four dialyses against phosphate-buffered saline (PBS). mIS200 sequence: _Acétyl+_C_76_MNNDDALAENNLKLPE_92_CY.

### Immunizations with mIS200 peptide

DBA/2 mice were immunized four times by intramuscular injections with mIS200 peptide (100 μg/mouse) or KLH alone (200 μg/mouse). The primo-injection was completed with complete Freund adjuvant (Sigma-Aldrich, Saint-Quentin Fallavier, France) and the three boosters, with an interval of 15 days, with incomplete Freund adjuvant (Sigma-Aldrich). Immunizations were performed 31, 17, and 3 days before the first injection of bleomycin and 11 days after the first injection of bleomycin.

### Mouse anti-IL-6 antibody ELISAs

Serum samples were obtained from mice at sacrifice, and the IgG response against mouse IL-6 was measured by enzyme-linked immunosorbent assay (ELISA). Briefly, 50 ng of mouse IL-6 (R&D Systems, Lille, France) was adsorbed on microtitration plates overnight. After a step of saturation, sera from immunized mice were serially diluted in PBS bovine serum albumin 1% and added in coated wells. A wash was followed by an incubation with polyclonal anti-mouse IgG as secondary antibodies coupled to horseradish peroxidase. Plates were revealed with tetramethylbenzidine, and the reaction was stopped with 1 M sulfuric acid before reading on a spectrophotometer (Multiskan Ex, Thermo Scientific) at 450 nm. ELISA titers were expressed as those serum dilutions that lead to half-maximal optical density 450 (OD_450_) (titer_50_).

### Evaluation of dermal and hypodermal thickness

Lesional skin areas were excised, fixed in 4% formalin, and embedded in paraffin. Sections (5 μm thick) were stained with hematoxylin and eosin. The dermal thickness was analyzed at 100-fold magnification by measuring the distance between the epidermal-dermal junction and the dermal-subcutaneous fat junction at four sites from lesional skin of each mouse. The hypodermal thickness in Tsk-1 mice was determined by measuring the thickness of the subcutaneous connective tissue beneath the panniculus carnosus at four different sites of the upper back in each mouse [8]. Two independent examiners performed the evaluation.

### Collagen measurements

The collagen content in lesional skin samples was evaluated by the hydroxyproline assay [24]. After digestion of punch biopsy specimens (3 mm diameter) in 6 M HCl for 3 hours at 120°C, the pH of the samples was adjusted to 7. Afterwards, samples were mixed with 0.06 M chloramine T and incubated for 20 minutes at room temperature. Then, 3.15 M perchloric acid and 20% p-dimethylaminobenzaldehyde were added, and samples were incubated for an additional 20 minutes at 60°C. The absorbance was determined at 557 nm with a spectrophotometer. For direct visualization of collagen fibers, trichrome staining was performed.

### Immunohistochemical analysis of α-SMA, CD3, CD22, IL-6, and IL-6-R

Myofibroblasts were identified by staining for α-smooth muscle actin (α-SMA), as previously described [8, 9, 21]. Cells positive for α-SMA in mouse skin sections were detected by incubation with monoclonal anti-α-SMA antibody (clone 1A4; Sigma-Aldrich, Saint-Quentin Fallavier, France) diluted 1:1,000 for 3 hours at room temperature. Polyclonal rabbit anti-mouse antibodies labeled with horseradish peroxidase (Dako, Les Ulis, France) were used as secondary antibodies for 1 hour at room temperature. The number of myofibroblasts was determined at 200-fold magnification in six different sections from each mouse by two blinded examiners.

To quantify the numbers of infiltrating T and B cells, lesional skin sections were stained for CD3 and CD22, respectively. Skin sections were incubated with polyclonal rabbit anti-human antibodies for CD3 or CD22 (Abcam, Paris, France). Polyclonal horseradish goat anti-rabbit antibodies labeled with horseradish peroxidase (Dako) were used as secondary antibodies. T and B cells were counted in a blinded manner, by two examiners, in six different sections of lesional skin of each mouse at 400-fold magnification.

To detect human IL-6 in lesional skin tissue, skin sections were incubated with monoclonal mouse anti-human antibodies against IL-6 (Abcam). Polyclonal rabbit anti-mouse antibodies labeled with horseradish peroxidase (Dako) were used as secondary antibodies. To detect mouse IL-6R in lesional skin tissue, polyclonal goat anti-mouse antibodies against IL-6R (R&D Systems) were used. The intensity of immunostaining was quantified with ImageJ software, as described in the following webpage [25].

### IL-6 measurement in lesional skin samples of bleomycin treated mice

IL-6 levels were measured in the skin of mice injected with bleomycin or NaCl by multiplex bead array technology (BD Biosciences, Le Pont de Claix, France), as previously described [8, 9].

### Serum levels of IL-6 and IL-6R

Serum concentrations of IL-6 (pg/mL) and IL-6R (pg/mL) were measured in a previously described cohort of 187 patients with SSc and 48 unrelated age/sex-matched subjects by quantitative sandwich ELISAs (R&D Systems, Minneapolis, MN, USA) in accordance with the recommendations of the manufacturer [26]. Serum levels of IL-6 were also measured in mice subjected to bleomycin injections and passive or active immunization as well as in their respective controls by using a U-cytech sandwich mouse IL-6 ELISA kit (U-cytech Biosciences, Utrecht, The Netherlands).

### Statistics

Results were expressed as dot blots with median_(Q1,Q3)_. Mann-Whitney *U* test for non-related samples was used for statistical analyses. *P* values of less than 0.05 were considered significant.

## Results

### Serum levels and skin expression of IL-6 are increased in patients with early systemic sclerosis

**Figure 1:**
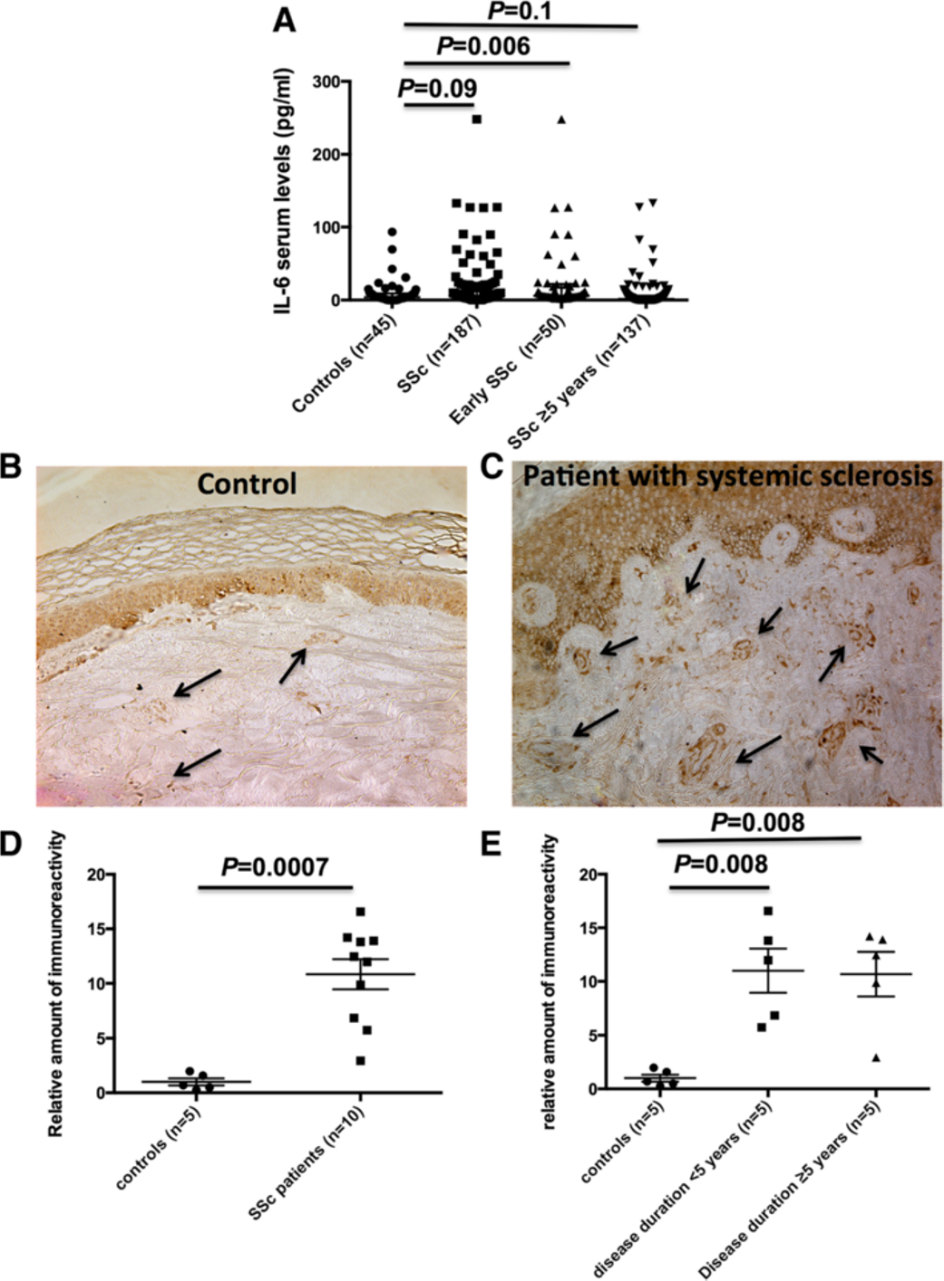
**Interleukin-6 (IL-6) is overexpressed in the serum and the skin of patients with systemic sclerosis (SSc). (A)** Patients with SSc and early disease (less than 5 years) had increased IL-6 serum levels compared with age- and sex-matched healthy controls (*P* = 0.006). IL-6 was detected *ex vivo* by immunohistochemistry in patients with SSc **(C)** compared with controls **(B)**. Positive staining for IL-6 was observed in the epidermis and in several cell types of the dermis. In addition, the intensity of immunostaining assessed by the ImageJ software was significantly increased in patients with SSc compared with controls (*P* = 0.0007) **(D)**, particularly in those with early disease (*P* = 0.008) **(E)**. Bars represent median_(Q1,Q3)_.

We first evaluated IL-6 and IL-6R expression in the serum of 187 patients with SSc as compared with 48 healthy controls. Higher median IL-6 serum concentrations were measured in patients with SSc compared with controls, although not reaching significant threshold (5.6 *versus* 4.0 pg/mL, *P* = 0.09) (Figure 1A). In the subset of patients with early disease (disease duration less than 5 years), median IL-6 serum levels were significantly higher than in controls (5.8 versus 4.0 pg/mL, *P* = 0.006) (Figure 1A). No difference was observed between SSc and controls regarding IL-6R serum levels (data not shown).

We next assessed skin expression of IL-6 by immunohistochemistry in patients with SSc and controls. Consistent with the results obtained in serum, we observed overexpression of IL-6 in the skin of patients with SSc, including those with early disease (disease duration of less than 5 years) (Figures 1B-E). The expression of IL-6 was detectable in the 10 patients with SSc and in the five controls. Positive staining for IL-6 was observed in the epidermis and in several cell types in the dermis, including perivascular inflammatory cells, fibroblasts, and endothelial cells (Figures 1B and 1C). Moreover, the amount of immunostaining was more abundant in patients with SSc compared with controls (*P* = 0.008), including those with early disease (*P* = 0.008) (Figures 1D and 1E). Taken together, these data support overexpression of IL-6 in patients with early SSc.

### MR16-1 prevents bleomycin-induced dermal fibrosis

**Figure 2:**
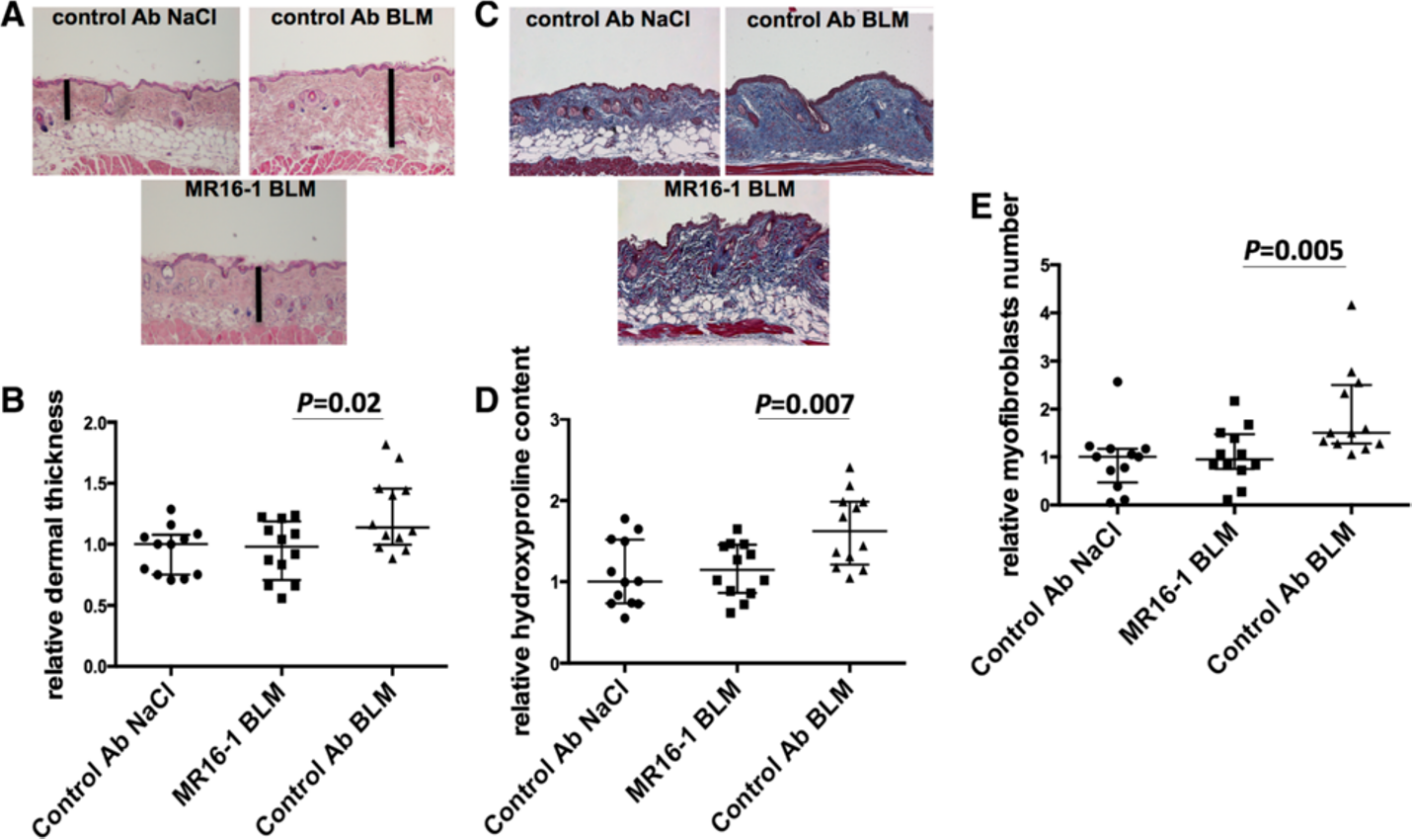
**Mice treated with MR16-1 are protected against bleomycin-induced dermal fibrosis. (A)** Reduced dermal fibrosis in mice treated with MR16-1, injected with bleomycin. Representative hematoxylin and eosin-stained skin sections are shown. **(B)** Decreased dermal thickness in mice treated with MR16-1 (change by median_(Q1,Q3)_ 1.0_0.7,1.2_ versus 1.1_1.0,1.5_ fold, *P* = 0.02). **(C)** Reduced accumulation of collagen in mice treated with MR16-1 following bleomycin treatment. Collagen fiber visualization by trichrome-staining skin sections is shown. **(D)** Reduced hydroxyprolin content in mice treated with MR16-1 following bleomycin treatment (change by median_(Q1,Q3)_ 1.1_0.9,1.4_ versus 1.6_1.2,2.0_ fold, *P* = 0.007). **(E)** Lower myofibroblast counts in mice treated with MR16-1 following bleomycin treatment (change by median_(Q1,Q3)_ 0.9_0.8,1.5_ versus 1.5_1.3,2.5_ fold, *P* = 0.005). Control mice were injected with NaCl, and the value for these mice was defined as 1; the results from the other groups were normalized to this value. Bars represent median_(Q1,Q3)_; 36 mice were used for these experiments (12 per group). Ab, antibody.

MR16-1 treatment prevented the induction of bleomycin-induced dermal fibrosis. Upon bleomycin injections, dermal thickness was reduced by 25% in mice treated with MR16-1 compared with mice treated with the isotype control (*P* = 0.02) (Figures 2A and 2B). Consistent with decreased dermal thickening, reduced accumulation of collagen upon bleomycin challenge was observed on trichrome-stained skin sections of mice treated with MR16-1 (Figure 2C). In addition, the hydroxyproline content in lesional skin was decreased by 30% in mice treated with MR16-1 compared with those treated with the isotype control (*P* = 0.007) (Figure 2D). The number of myofibroblasts upon challenge with bleomycin was also significantly reduced by 45% in mice treated with MR16-1 (*P* = 0.005) (Figure 2E).

### MR16-1 exerts no antifibrotic effect in Tsk-1 mice

**Figure 3:**
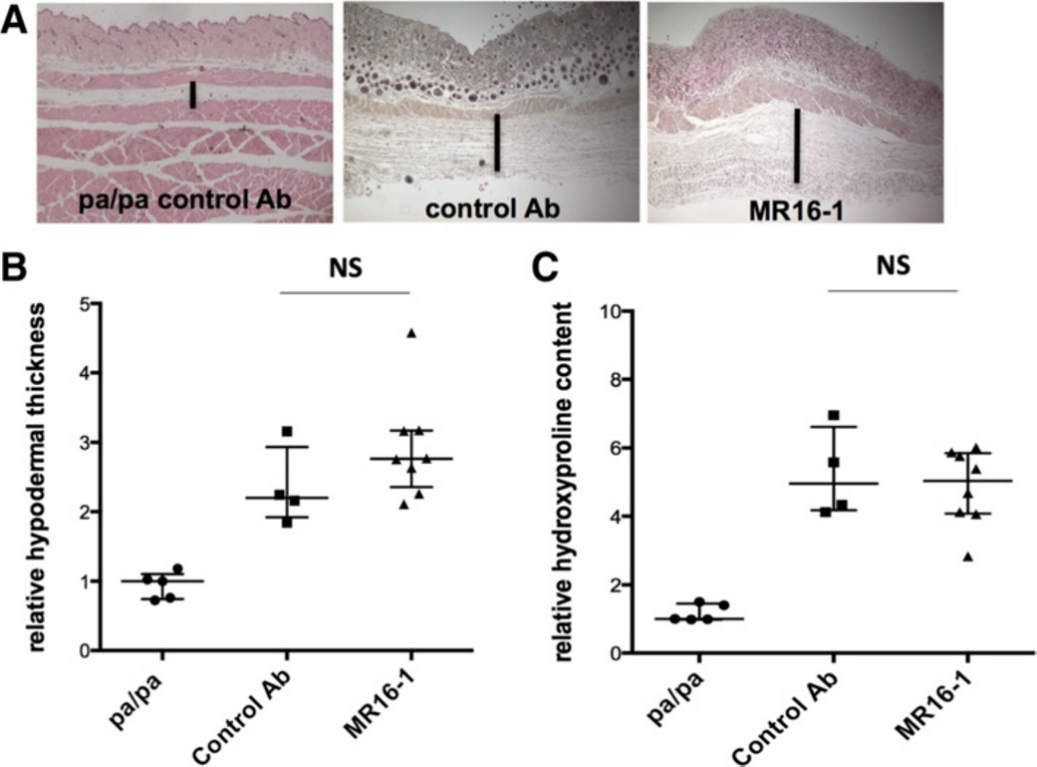
**Tight skin-1 (Tsk-1) mice are not protected from skin fibrosis by MR16-1 treatment. (A, B)** Tsk-1 mice showed increased hypodermal thickness compared with the pa/pa control mice. However, no difference was observed between Tsk-1 mice receiving either MR16-1 or the isotype control. **(C)** Tsk-1 mice showed increased hydroxyproline concentration compared with the pa/pa control mice. However, no difference was observed between Tsk-1 mice receiving either MR16-1 or the isotype control. pa/pa mice (control mice for Tsk-1 mice) were defined as 1.0, and all other results are normalized to this value. Bars represent median_(Q1,Q3)_; 17 mice were used for these experiments (five pa/pa mice, four Tsk-1 mice treated with isotype control, and eight Tsk-1 mice treated with MR16-1). Ab, antibody; NS, not significant.

We next investigated whether treatment with MR16-1 may also be effective in a non-inflammatory model of SSc. We observed that Tsk-1 mice treated with MR16-1 showed no reduction of hypodermal thickness (Figures 3A and 3B) and collagen content (Figure 3C) compared with those receiving the isotype control.

### Active immunization against IL-6 in the bleomycin mouse model

#### Immunization of mice with mIS200 peptide is well tolerated

Following the encouraging results obtained with MR16-1 in the prevention of bleomycin-induced dermal fibrosis, we aimed to evaluate the protective effect of an anti-IL-6 peptide-based active immunization in this model. Immunization of mice with mIS200 peptide at a dose of 100 μg per injection was well tolerated. mIS200-immunized mice appeared healthy with normal activity, behavior, and texture of the fur. The body weight also did not differ between mice treated with mIS200 and KLH-immunized control.

#### Anti-IL-6 autoantibody production

**Figure 4:**
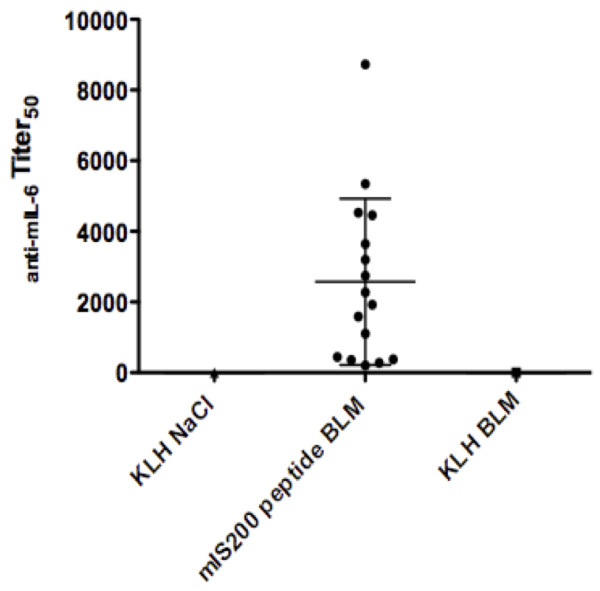
**Anti-mIL-6 autoantibody production in the group of mice immunized against mIS200 peptide and in the groups of mice immunized against keyhole limpet hemocyanin (KLH) alone.** No anti-IL-6 autoantibodies were detected in serum samples from mice immunized against KLH alone compared with the mice immunized against mIS200 peptide. Bars represent mean ± standard deviation.

DBA/2 mice were immunized four times with mIS200 peptide, and bleomycin-induced fibrosis was induced after the second booster injection. Anti-IL-6 antibody production was evaluated by ELISA at sacrifice. Mice immunized against mIS200 peptide produced a high level of autoantibodies against IL-6, whereas mice immunized against KLH alone did not exhibit anti-IL-6 antibodies (Figure 4). All mice showed antibody response to the carrier protein KLH (data not shown).

#### Anti-IL-6 peptide-based active immunization alleviates bleomycin-induced skin fibrosis

**Figure 5:**
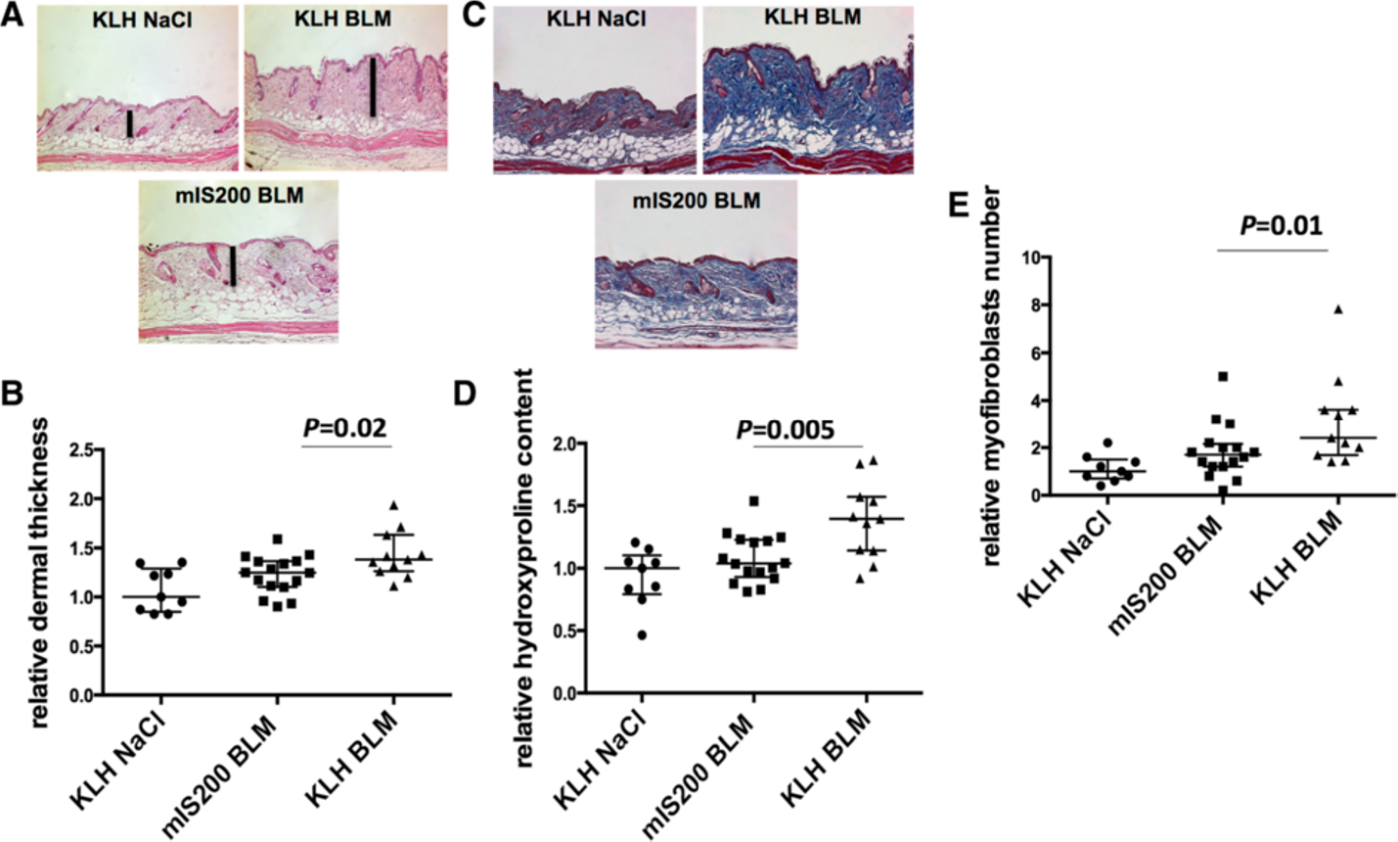
**Mice immunized against mIS200 peptide are protected against bleomycin-induced dermal fibrosis. (A)** Reduced dermal fibrosis in mice immunized against mIS200 peptide injected with bleomycin. Representative hematoxylin and eosin-stained skin sections are shown. **(B)** Decreased dermal thickness in mice immunized against mIS200 peptide treated with bleomycin (change by median_(Q1,Q3)_ 1.2_1.1,1.4_ versus 1.4_1.3,1.6_ fold, *P* = 0.02). **(C)** Reduced accumulation of collagen in mice immunized against mIS200 peptide challenged with bleomycin. Collagen fiber visualization by trichrome-staining skin sections is shown. **(D)** Reduced hydroxyprolin content in mice immunized against mIS200 peptide following bleomycin treatment (change by median_(Q1,Q3)_ 1.0_0.9,1.2_ versus 1.4_1.1,1.6_ fold, *P* = 0.005). **(E)** Lower myofibroblast counts in mice immunized against mIS200 peptide following bleomycin treatment (change by median_(Q1,Q3)_ 1.7_1.2,2.2_ versus 2.4_1.7,3.6_ fold, *P* = 0.01). Control mice were injected with NaCl, and the value for these mice was defined as 1; the results from the other groups were normalized to this value. Bars represent median_(Q1,Q3)_; 36 mice were used for these experiments (nine in the group keyhole limpet hemocyanin (KLH) NaCl, 16 in the group mIS200 bleomycin, and 11 in the group KLH bleomycin).

Mice immunized against the mIS200 peptide exhibited a significant reduction of dermal thickness by 20% compared with the group immunized against KLH alone (*P* = 0.02) (Figures 5A and 5B). Consistently with decreased dermal thickening, a significant reduction of the collagen content following bleomycin challenge was observed on trichrome-stained skin sections from mice immunized against mIS200 peptide (Figure 5C). Furthermore, the hydroxyproline content in the lesional skin of mIS200-immunized mice was significantly decreased by 25% compared with the skin of KLH-immunized mice (*P* = 0.005) (Figure 5D). In addition, the number of myofibroblasts was decreased by 41% in mIS200-immunized mice (*P* = 0.01) (Figure 5E).

### Passive or active immunization against IL-6 reduce T-cell infiltration in lesional skin

**Figure 6:**
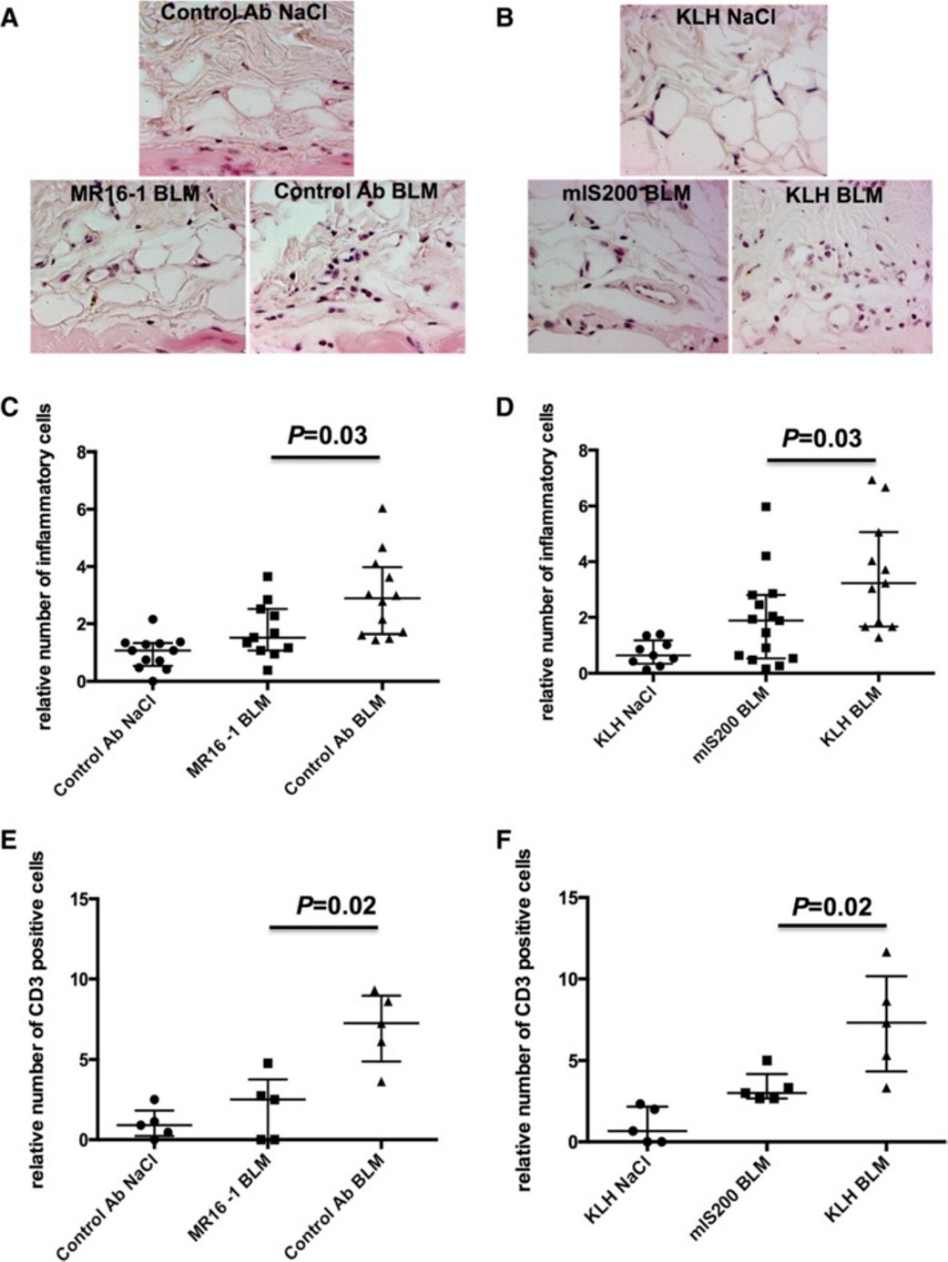
**Interleukin-6** **(IL-6) regulates leukocyte and T-cell infiltration into lesional skin. (A, B)** Reduced inflammation in mice treated with MR16-1 or immunized against the mIS200 peptide and challenged with bleomycin. Representative sections stained by hematoxylin/eosin at 400-fold magnification. **(C, D)** Decreased leukocyte counts in lesional skin of mice treated with MR16-1 or immunized against the mIS200 peptide. **(E, F)** Reduced number of CD3^+^ T cell by immunohistochemistry in lesional skin of mice treated with MR16-1 or immunized against the mIS200 peptide. Bars represent median_(Q1,Q3)_; in regard to leukocyte quantification, 36 mice were evaluated for passive immunization and 36 for active immunization. In regard to T-cell quantification, 15 randomly chosen mice (five per group) were evaluated for passive immunization as well as for active immunization. All results are normalized to mice injected with NaCl. Ab, antibody; KLH, keyhole limpet hemocyanin.

Inflammatory infiltrates are characteristic features of early stages of SSc, which are mimicked in the bleomycin-induced fibrosis mouse model. Infiltrating leukocytes contain mostly T cells, with a perivascular distribution, and stimulate fibroblast activation and collagen synthesis via the release of pro-fibrotic factors [1]. To evaluate whether passive or active immunization against IL-6 influences the outcome of bleomycin-induced fibrosis by regulating leukocyte infiltration, we quantified the number of leukocytes in lesional skin. Inflammatory infiltrates upon bleomycin treatment were significantly reduced in mice treated with MR16-1 or immunized against the mIS200 peptide compared with mice injected with isotype control or KLH (*P* = 0.03 for both comparisons) (Figures 6A-D). Since T cells are the main component of inflammatory infiltrates, we quantified the number of T cells in fibrotic skin. Consistent with the reduced number of leukocytes, T-cell counts were significantly lower in mice treated with MR16-1 or immunized against the mIS200 peptide compared with their respective control groups (*P* = 0.02 for both comparisons) (Figures 6E and 6F). In contrast to T cells, the number of B cells did not significantly differ upon bleomycin challenge between mice treated with MR16-1 or immunized against the mIS200 peptide and those injected with isotype control or KLH (data not shown).

### Serum and skin levels of IL-6 are regulated by passive or active immunization against IL-6

**Figure 7:**
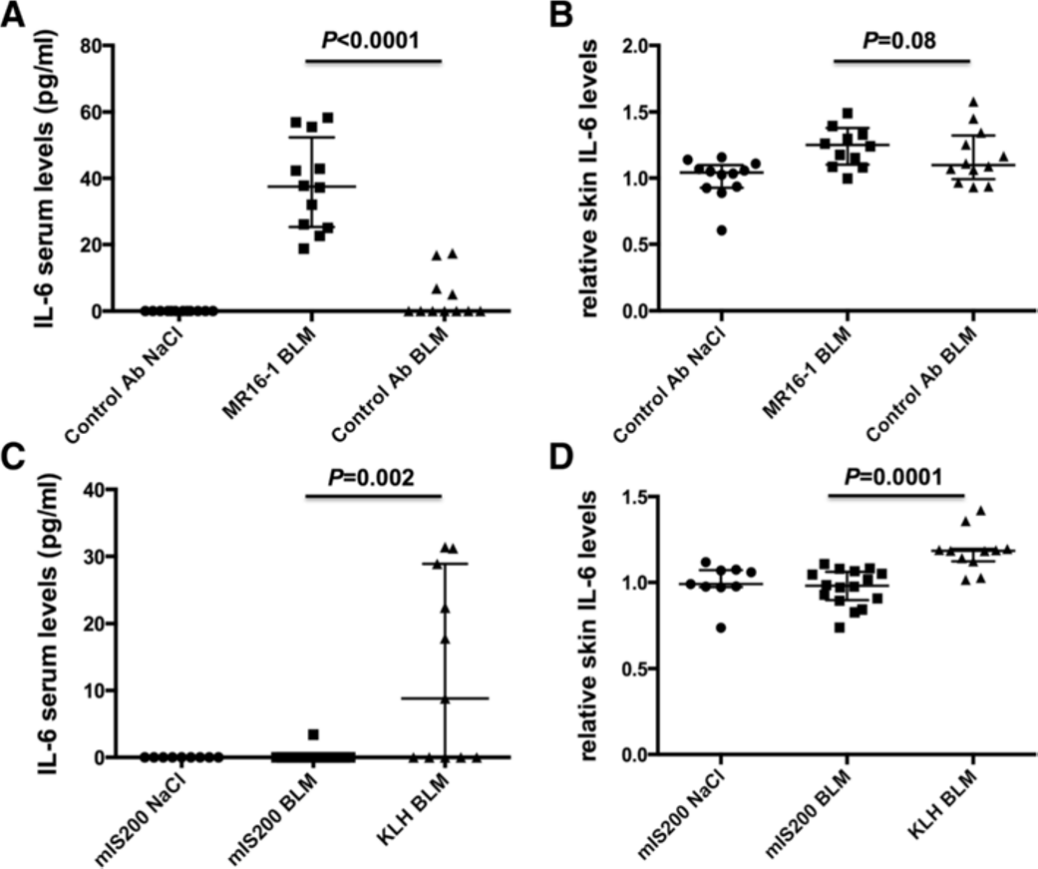
**Passive and active immunizations against interleukin-6 (IL-6) regulate serum levels and skin expression of IL-6. (A, B)** In mice challenged by bleomycin, MR16-1 treatment led to a significant increase of IL-6 serum levels (*P* <0.0001) and a trend for increased IL-6 skin expression (*P* = 0.08). **(C, D)** Immunization against the mIS200 peptide conducted to a significant reduction of IL-6 in the serum and in the skin of mice challenged with bleomycin. Bars represent median_(Q1,Q3)_; in total, 36 mice were evaluated for passive immunization and 36 for active immunization. All results are normalized to mice injected with NaCl.

To better apprehend the effects of passive and active immunization in the bleomycin-induced dermal fibrosis mouse model, IL-6 and IL-6R expressions were assessed in the lesional skin of mice by multiplex bead array technology and immunohistochemistry, respectively. In addition, serum levels of IL-6 were measured by ELISA. Mice treated with MR16-1 and subjected to bleomycin injections displayed significantly higher IL-6 serum levels than mice receiving the isotype control (Figure 7A), as reported in previous publications [27]. Consistent with this result, a trend for higher skin levels of IL-6 was observed upon MR-16 treatment (Figure 7B). Upon bleomycin challenge, IL-6 levels were significantly reduced in the serum and the skin of mice immunized against the mIS200 peptide compared with mice injected with KLH (Figures 7C and 7D).

Dermal expression of IL-6R was not modified by passive or active immunization against IL-6 in mice challenged with bleomycin injections (data not shown).

## Discussion

We first confirmed that IL-6 is overexpressed in the serum and the skin of patients with SSc and particularly in those with early disease. Since upregulation of serum IL-6 has been shown to be associated in SSc with disease activity, severity, disability, worse outcome, and reduced survival, targeting IL-6 may be particularly relevant in patients with early disease. Our results in mice also confirm the relevance of targeting IL-6 in early SSc since IL-6 exhibits a critical role in the development of bleomycin-induced dermal fibrosis, which reflects early and inflammatory stages of SSc. Indeed, inhibition of IL-6 through an innovative anti-IL-6 active immunization strategy exerted similar antifibrotic properties as passive immunization with MR16-1 (monoclonal antibody against IL-6-R) in the bleomycin-induced dermal fibrosis mouse model, by reducing the infiltration of inflammatory cells, especially T cells, into lesional skin. Conversely, MR16-1 did not prevent the development of fibrosis in the Tsk-1 mouse model, suggesting that IL-6 has no direct effects on fibroblast activation and collagen synthesis in this model of late and non-inflammatory stages of SSc.

We first performed passive immunization with MR16-1 in the bleomycin mouse model (i) to confirm previously reported findings in our in-house bleomycin-challenged mice and (ii) to obtain a range of effect of passive immunization efficacy, allowing further comparison with active immunization. We showed that mice treated with MR16-1 were protected from the development of dermal fibrosis, with a significant reduction of dermal thickness, collagen content, and infiltrating myofibroblasts in the lesional skin. Our results were in line to those of Kitaba *et al.* [13], despite several differences in the experiments performed. In particular, we used DBA/2 mice in our study, which show higher susceptibility to bleomycin-induced fibrosis than C57BL/6 mice [28]. Both studies differed for the bleomycin injection protocol (bleomycin at a concentration of 0.5 mg/mL injected subcutaneously every other day for 3 weeks in our study compared with a concentration of 1 mg/mL injected subcutaneously daily for 4 weeks). We have used this protocol in several previous projects [8, 9, 21]. The treatment scheme with MR16-1 was also different between both studies (a first i.p. injection of 2 mg at day 0 then i.p. injections of 1 mg at days 7 and 14 in our study compared with a first intravenous injection of 2 mg at day 0 then i.p. injections of 0.5 mg at days 7, 14, and 21) [13]. Our data showed that MR16-1 exerts antifibrotic effects in the model of bleomycin-induced skin fibrosis by reducing the infiltration of T cells into lesional skin, which was not reported before. Reduced T-cell infiltration may lead to decreased resident fibroblast activation in lesional skin. This is consistent with the reduction of myofibroblast counts in MR16-1-treated mice, upon bleomycin challenge, observed in our study and previously reported by Kitaba *et al.* [13].

We also observed a significant increase of serum IL-6 levels and a trend for increased skin IL-6 expression in mice treated with MR16-1. This is in line with previous observations of increased blood IL-6 levels after anti-IL-6R antibody injection [27]. This elevation seems to be the result of IL-6 clearance inhibition due to IL-6R blockade, rather than induction of IL-6 synthesis to counterbalance IL-6 blockade, or release of free IL-6 from complexes [27].

Since previous studies focused on the effects of IL-6 inhibition with MR16-1 in mouse models of bleomycin-induced dermal fibrosis, we next aimed to assess the effect of MR16-1 in a non-inflammatory mouse model of SSc. Thus, we investigated the effects of MR16-1 in the Tsk-1 mouse model, in which increased serum IL-6 levels have been reported [7]. However, MR16-1-treated mice did not exhibit a reduction of skin fibrosis, suggesting that other pathways or cytokines may be more important in this specific mouse model. This result also supports that IL-6 exerts indirect profibrotic effects in early inflammatory stages of SSc rather than by direct effects on the collagen synthesis by fibroblasts. This result contrasts with the direct profibrotic effects of IL-6 observed recently on dermal fibroblasts [29]. Since these results were obtained *in vitro* in human fibroblasts, they may differ from those obtained *in vivo* in the very specific Tsk-1 mouse model. The absence of antifibrotic effects of MR16-1 in the Tsk-1 mice is also sustained by the controversial role of immune cells, especially T and B cells, in the development of fibrosis in this mouse model. Although a role for CD4^+^ T and B cells has been suggested in the activation of collagen synthesis, bone marrow transplantation experiments have challenged the contribution of these immune cells [7, 30]. Indeed, the transfer of enriched B or T cells increased autoantibody production but did not cause skin fibrosis, and transfer of T and B lymphocytes led only to mild fibrotic lesions compared with massive fibrosis in Tsk-1 mice [31]. In line with this finding, Tsk-1 mice lacking mature T and B lymphocytes developed a fibrotic phenotype in the absence of a functional immune system [32]. Taken together, our results support that passive immunotherapy with MR16-1 has interest only in inflammation-driven dermal fibrosis; thus, this strategy should preferentially be tested in early inflammatory stages of SSc.

Following our results obtained with MR16-1 in the bleomycin mouse model, we evaluated the antifibrotic effects of IL-6 inhibition with an innovative anti-cytokine strategy. Since the late ’90s, anti-cytokine biologics (monoclonal antibodies and soluble receptors) have successfully been used to treat chronic inflammatory diseases. However, these biotherapies present several drawbacks, including primary and secondary resistances, repeated injections, side effects, and prohibitive costs. To circumvent these drawbacks, new anti-cytokine strategies, such as the anti-cytokine active immunization strategy, are in development [33]. This is notably highlighted by the use of anti-TNFα kinoid in a phase-2 trial in rheumatoid arthritis (ClinicalTrials.gov identifier NCT01040715) and Crohn’s disease (ClinicalTrials.gov identifier NCT01291810). In the present study, mice immunized against an IL-6 peptide exhibited a significant reduction of dermal fibrosis, collagen content, and myofibroblasts in the bleomycin mouse model with no adverse event. Moreover, this prevention was similar to the one conferred by the MR16-1 monoclonal antibody (25% and 20% reduction of dermal thickness, respectively, and 25% and 30% reduction of hydroxyproline content, respectively). Our results suggest that active immunization against IL-6 displays antifibrotic properties by decreasing T-cell infiltration into lesional skin, similarly as passive immunization, and by reducing IL-6 levels, as demonstrated by decreased IL-6 levels in the serum and the skin of mice immunized against the mIS200 peptide and challenged with bleomycin. This work shows the feasibility and the efficacy of targeting IL-6 by a peptide-based active immunization in order to block endogenous IL-6 pathological effects. Of note, this strategy was not evaluated in the Tsk-1 mouse model regarding the negative results obtained with the passive immunization strategy.

Evaluation of passive and active immunization was not evaluated to reverse established fibrosis, which is a limitation of our study. Kitaba *et al.* [13] have previously shown that passive immunization with MR16-1 improves established bleomycin-induced dermal fibrosis. Further studies are required to assess whether active immunization may be curative in the modified bleomycin model of established dermal fibrosis.

## Conclusions

Using complementary mouse models of SSc, we demonstrated that passive and active immunization targeting IL-6 had similar antifibrotic effects in the mouse model of bleomycin-induced dermal fibrosis, which are mediated by the reduction of T-cell infiltration into lesional skin and by the decreased skin IL-6 levels in the case of active immunization. Translation to human disease is now required, and targeting early and inflammatory stages of SSc sounds the most appropriate. The passive immunization strategy is under investigation in a phase-3 clinical trial assessing the efficacy of tocilizumab to improve skin involvement in patients with early diffuse SSc (ClinicalTrials.gov identifier NCT01532869). Targeted innovative therapies are the most important issue in SSc, which is a very severe condition free of an efficient drug. It is awaited eagerly by the whole medical community, together with the patients, in order to stop the progression of tissue damage by this devastating disease.

Our results also highlight the relevance of anti-IL-6 peptide-based active immunotherapy to treat autoimmune diseases. Further investigations are needed to translate this strategy into humans, but it constitutes a promising therapeutic approach.

## Abbreviations

α-SMA: α-smooth muscle actin
BDB: bis-diazobenzidine
ELISA: enzyme-linked immunosorbent assay
IL: interleukin
IL-6R: interleukin-6 receptor
i.p.: intraperitoneal
KLH: keyhole limpet hemocyanin
PBS: phosphate-buffered saline
PDB: Protein Data Bank
SSc: systemic sclerosis
TNFα: tumor necrosis factor-alpha
Tsk-1: tight skin-1.
